# Generation of Reference Softgauges for Minimum Zone Fitting Algorithms: Case of Aspherical and Freeform Surfaces

**DOI:** 10.3390/nano11123386

**Published:** 2021-12-14

**Authors:** Amine Chiboub, Yassir Arezki, Alain Vissiere, Charyar Mehdi-Souzani, Nabil Anwer, Bandar Alzahrani, Mohamed Lamjed Bouazizi, Hichem Nouira

**Affiliations:** 1 Laboratoire Commun de Métrologie (LNE-CNAM), 1 Rue Gaston Boissier, 75015 Paris, France; amine.chiboub@external.hec.fr (A.C.); yassir.arezki@gmail.com (Y.A.); alain.vissiere@lecnam.net (A.V.); 2 Université Paris-Saclay, Université Sorbonne Paris Nord, ENS Paris-Saclay, LURPA, 91190 Gif-sur-Yvette, France; charyar.souzani@ens-paris-saclay.fr (C.M.-S.); nabil.anwer@ens-paris-saclay.fr (N.A.); 3 Department of Mechanical Engineering, College of Engineering, Prince Sattam bin Abdulaziz University (PSAU), Alkharj 16273, Saudi Arabia; ba.alzahrani@psau.edu.sa (B.A.); my.bouazizi@psau.edu.sa (M.L.B.)

**Keywords:** fitting algorithm validation, reference softgauges generation, minimum zone fitting, Chebyshev fitting, complex surfaces

## Abstract

Optical aspherical lenses with high surface quality are increasingly demanded in several applications in medicine, synchrotron, vision, etc. To reach the requested surface quality, most advanced manufacturing processes are used in closed chain with high precision measurement machines. The measured data are analysed with least squares (LS or L_2_-norm) or minimum zone (MZ) fitting (also Chebyshev fitting or L_∞_-norm) algorithms to extract the form error. Performing data fitting according to L_∞_-norm is more accurate and challenging than L_2_-norm, since it directly minimizes peak-to-valley (PV). In parallel, reference softgauges are used to assess the performance of the implemented MZ fitting algorithms, according to the *F1 algorithm measurement standard*, to guarantee their traceability, accuracy and robustness. Reference softgauges usually incorporate multiple parameters related to manufacturing processes, measurement errors, points distribution, etc., to be as close as possible to the real measured data. In this paper, a unique robust approach based on a non-vertex solution is mathematically formulated and implemented for generating reference softgauges for complex shapes. Afterwards, two implemented MZ fitting algorithms (HTR and EPF) were successfully tested on a number of generated reference pairs. The evaluation of their performance was carried out through two metrics: degree of difficulty and performance measure.

## 1. Introduction

Conformance assessment of manufactured parts to design tolerance specification is a major activity in the quality control process. Traceable ultra-high precision coordinate measuring machines (CMMs) to the SI metre definition are usually deployed to generate a set of data points lying on the artefact’s surface [1]. The recorded data will be processed in order to infer information about the measured surface. This task becomes difficult when it comes to the measurement of aspherical and freeform surfaces due to their complex geometry. Different factors could greatly affect the machine uncertainty, namely, the work piece, the hardware of the CMMs, the sampling strategy, the fitting algorithms, etc. [1,2,3]. Fitting could be defined as the process of determining parameters of the geometric features that best describe the measured data according to a defined criterion. This geometric element is called “*associated feature*” [4]. Different criteria could be used, including least squares (LS) (or Gaussian, or L_2_-norm fitting), minimum zone (MZ) (or minimax, Chebyshev, L_∞_-norm fitting) and one-sided measures, such as minimum circumscribed (MC) or maximum inscribed (MI) elements. While the two least criteria are well adapted for circular or cylindrical features, the former ones could be applied for almost all shapes. Least squares criterion, which is more adapted when random measurement errors predominate, originates from maximum likelihood theory [5]. It is the most widely used criterion in industry and most fitting commercial software relies on it. However, in the accept/reject process, LS fitting may not give complete information and may cause the rejection of conforming parts. Therefore, the use of MZ fitting reflects the functional requirements of measured parts. This criterion is appropriate when measuring errors are small compared to manufacturing ones. Moreover, MZ is considered as the default fitting criterion in the ISO geometrical product specification (GPS) [6,7]. Once the MZ value is found, a measure of variability must be set and the part will be accepted if the measure is below design limits. The commonly used measure is the peak-to-valley (PV), which is the difference between maximum and minimum form deviations. Unlike LS fitting methods, the implementation of MZ fitting methods is challenging, since it directly minimizes the peak-to-valley (PV) [8].

For metrology of aspherical and freeform surfaces, a few reference fitting algorithms have recently been developed. Key characteristics of reference fitting algorithms were outlined in [8]. Thus, it is stated that they must:

- perform well for representative data
- work sensibly for unrepresentative data and be able to detect extreme cases
- perform efficiently in poor cases

Despite its importance, performance in terms of execution time is not the first characteristic sought for reference MZ fitting algorithms. However, as reported in [2] the algorithm must be stable and robust. The stability means that the underlying numerical operations are numerically stable. For example, small perturbations in input data must only result in small perturbations in output. Robustness refers to the algorithm ability to handle extreme cases.

To establish the traceability chain for datasets analysis with a small uncertainty (below the nanometre level), the validation of implemented reference MZ fitting algorithms becomes indispensable in order to make sure that the returned values are correct. There exist two methods to assess the correctness of the values returned by MZ fitting algorithms [9,10,11,12], namely *type F1 algorithm measurement standard* (using reference softgauges pair) and *type F2 algorithm measurement standard* (using reference algorithm) [11].

For complex geometries, an approach based on *type F1* was presented in [13]. However, the proposed method generates reference softgauges with vertex solution only. Meanwhile, non-vertex solution occurs in practice as reported in [1,14].

In this article, the validation of MZ fitting algorithms using reference softgauges is discussed. Section 2 introduces the MZ fitting optimisation problem, and Section 3 investigates the validation procedures. The problem is mathematically formulated in Section 4, while a general approach is given in Section 5 on the generation of reference softgauges based on a non-vertex solution for the case of complex shapes. Numerical validation on two implemented fitting algorithms (exponential penalty function (EPF) and hybrid trust region (HTR)) is carried out in Section 6. Two metrics to determine the degree of difficulty and the performance measure are given in Section 7, so as to assess the performance of MZ fitting algorithms.

## 2. Data Fitting

**Figure 1:**
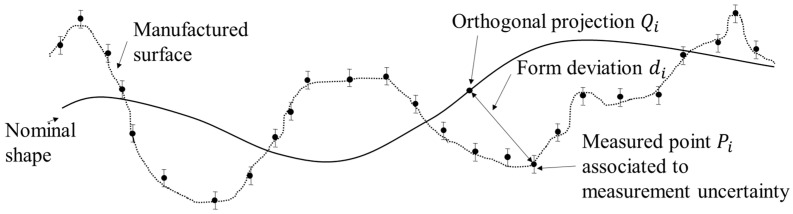
Definition of the form errors (or deviation errors).

LS or MZ fitting are considered as an optimisation problem. Given a set of N data points, ${\left\{P_{i}\right\}}_{1\leq i\leq N}$, let $f\left(\hat{X},s\right)=0$ is the generic equation describing the shape of the measured surface where $\hat{X}$ is the space vector ($\hat{X}=\left(\hat{x},\hat{y},\hat{z}\right)$ in Cartesian coordinates) and s denotes the shape parameters. For each point$\;P_{i}$, one could associate its orthogonal projection on the nominal surface denoted by $Q_{i}$. $d_{i}=\|P_{i}-Q_{i}\|$, where ‖.‖ is the Euclidean norm, which defines the orthogonal distance between the measured data points $P_{i}$ and the nominal surface, known as form deviation (Figure 1).

The objective function to minimize for LS (resp. MZ) is given in (1) (resp. (2)). $x=\left(m,\;s\right)\in {\mathbb{R}}^{n}$ could be either the set of intrinsic shape parameters s, the motion parameters m: rotation and translation applied to $\left\{P_{i}\right\}$, or both.

(1) $$\underset{x}{min}\overset{N}{\underset{i=1}{\sum }}{\left(\|P_{i}-Q_{i}\|\right)}^{2}\;$$

(2) $$\underset{x}{min}\underset{1\leq i\leq N}{max}\|P_{i}-Q_{i}\|$$

The formulations (1) and (2) represent different mathematical properties. Those problems were extensively studied for simple geometries and a number of techniques were developed, as reported in [15,16,17,18]. Existing methods like variants of the Gauss–Newton [8] could be applied to solve the smooth least squares objective function. The objective function in the second MZ problem (2) is not differentiable; thus, a number of differentiable optimization techniques cannot be applied. Numerous MZ fitting methods were suggested for the case of straightness, flatness, roundness and cylindricity tolerances [19,20,21]; however, rare methods were proposed for the MZ fitting of complex shapes. Nevertheless, some attempts were made in [22,23,24], where techniques such as smoothing functions, primal-dual interior point methods or differential evolution algorithms were adapted to aspherical shapes.

## 3. Validation of MZ Fitting Algorithms

**Figure 2:**
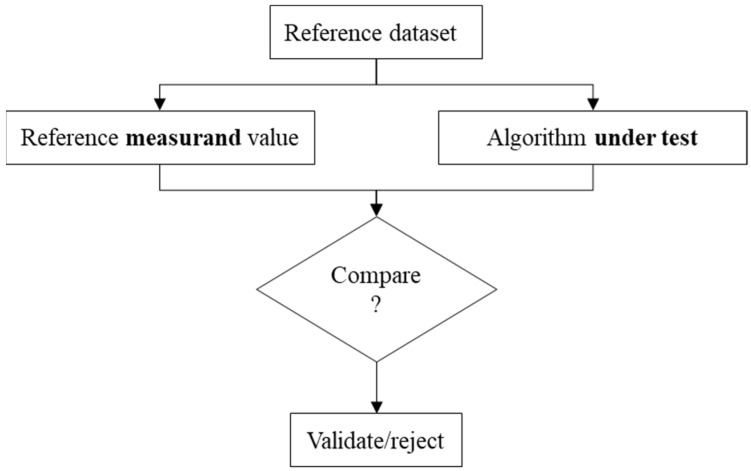
Illustration of the type F1 algorithm measurement standard.

The need for fitting algorithm validation in metrology was initiated and supported in [3,12]. The aim is to ensure that MZ fitting algorithms return correct values. One methodology to assess the results returned by metrology algorithms is to use a reference pair. This methodology known as “*Type F1 algorithm measurement standards”* is defined in ISO 5436-2 2012 [11]. Even if this *Type F1 standard* is common in surface texture domain, its concept could be extended to MZ fitting algorithms. *Type F1 standards* could be considered as a numerical representation of the measured part to which we associate a reference measure and value known with a given uncertainty. For the evaluation, reference softgauges are inputted to the metrology algorithm under test, the returned value is compared to the reference measure, and then a decision could be made as to whether the metrology algorithm is accepted or rejected (Figure 2).

**Figure 3:**
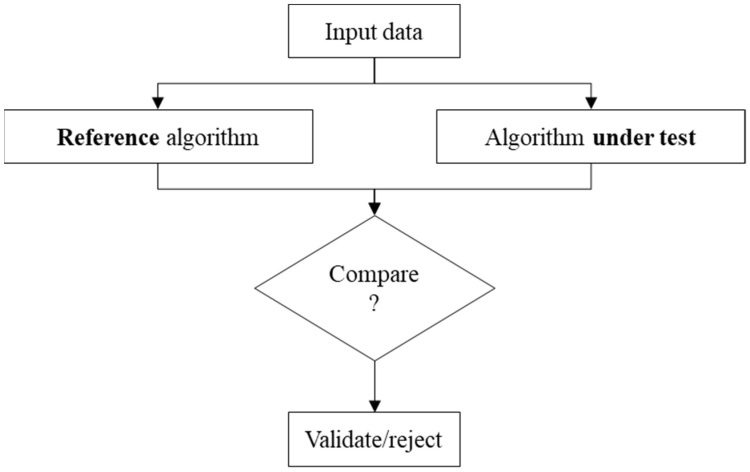
Illustration of the type F2 algorithm measurement standard.

A second methodology to evaluate metrology algorithms is based on the use of reference algorithms defined in ISO 5436-2 2012 as “*Type F2 algorithm measurement standards”* [11]. There are traceable metrology algorithms against which the tested algorithm will be compared. A common set of data points is submitted to both metrology algorithms (reference algorithm and algorithm under test) and the two results are then compared in order to take an accept/reject decision (Figure 3). Reference algorithms do not exist for a wide range of applications in metrology. Their development is not always straightforward, especially for applications such as MZ fitting.

**Figure 4:**
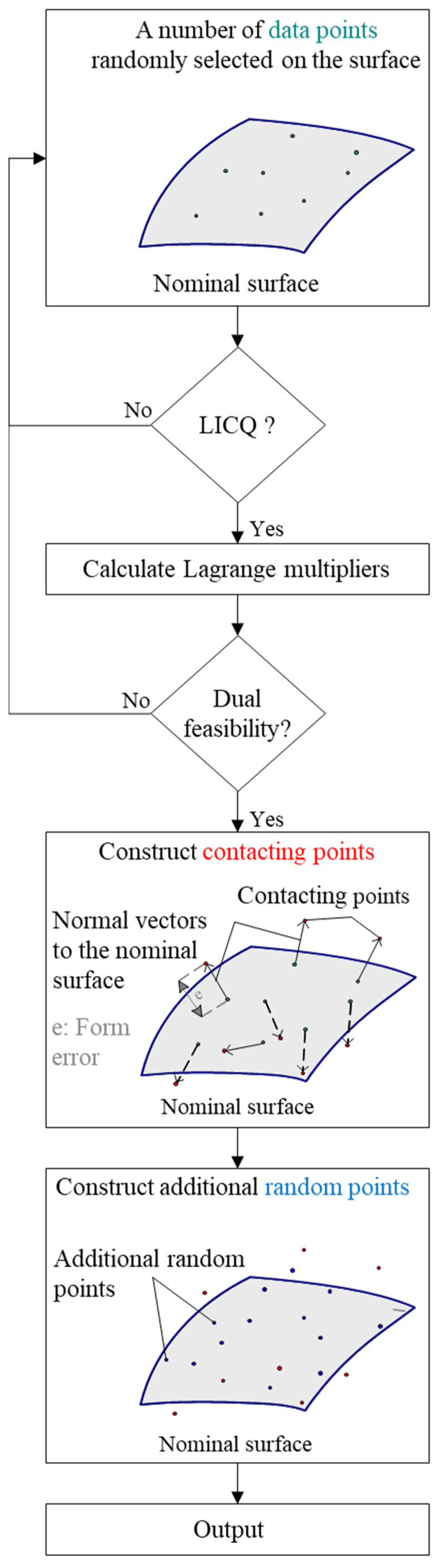
Flowchart describing the generation of reference softgauges for MZ fitting.

For MZ fitting, Forbes et al. [1] developed a method for the generation of reference softgauges with vertex solution. Similarly to LS fitting, the Karush–Kuhn–Tucker (KKT) optimality conditions for the MZ fitting problem are indispensable and the data points are generated such as to meet the number of conditions reported by Boyd et al. [25]. A set of points are selected on the nominal shape, such that the linear independence constraint qualification (LICQ) of the problem holds [26]. Then, equations resulting from KKT conditions are solved in order to determine the Lagrangian multiplier [25]. If the resulting Lagrange multipliers are not positive (dual feasibility does not hold), a new set of data points are chosen and the procedure is repeated. Once the dual feasibility condition is satisfied, the contacting points are constructed and additional random points are generated on the surface. A flowchart describing the proposed approach is illustrated in Figure 4.

## 4. Mathematical Formulation of the Reference Softgauges Generation for MZ Fitting

**Figure 5:**
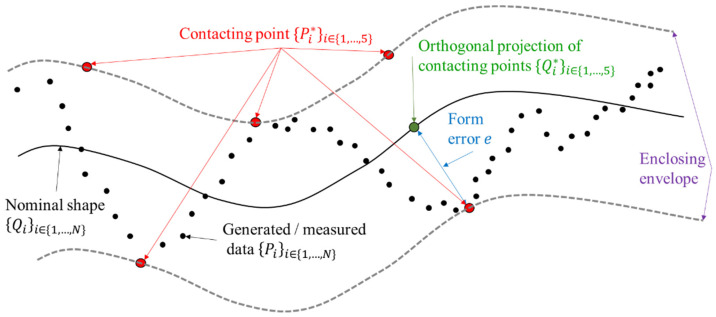
Illustration of five contacting points to the enclosing envelope. Three located on the upper bound and two on the lower bound.

Based on Figure 5, MZ fitting is formulated as a nonlinear programming (3).

(3) $$\underset{x,e}{min}\;e\;\mathrm{such}\mathrm{that}:-e\leq d_{i}\left(x\right)\leq e\;\forall i\in \left\{1,\ldots ,N\right\}$$

where e≥0 is the form error, ***N*** the number of measured points and $\;d_{i}=\|P_{i}-Q_{i}\|$.

The problem (3) could be formulated in the standard form given in (4).

(4) $$\underset{y}{min}\;f\left(y\right)\;\mathrm{subject}\mathrm{to}\;C_{i}\left(y\right)=\{\begin{array}{c} C_{i}^{+}\left(y\right)\geq 0\\ C_{i}^{-}\left(y\right)\geq 0 \end{array}\;\forall i\;\in \left\{1,\;\ldots ,N\right\}$$

where $y=\left(e,x\right)\in {\mathbb{R}}^{n+1}$, $C_{i}^{+}\left(y\right)=e-d_{i}\left(x\right)$, $C_{i}^{-}\left(y\right)=e+d_{i}\left(x\right)$ and f(y)=e.

Let $y^{*}$ be a local minimum of the problem (4). Hence,$\;y^{*}$ could be feasible when it satisfies the condition $C_{i}\left(y^{*}\right)\geq 0,\;\forall i\;\in \left\{1,\;\ldots ,N\right\}$.

At a feasible point $y^{*}$,

(5) $$\{\begin{array}{c} C_{i}\left(y^{*}\right)=0\;\;\;i^{th}\;constraint\;is\;active\\ \;\;C_{j}\left(y^{*}\right)>0\;\;\;j^{th}\;constraint\;is\;inactive \end{array}$$

An active constraint could be interpreted as a point belonging to the measured data for which the distance to the reference surface is equal to the form error (“*a contacting point to the enclosing envelope*”) as illustrated in Figure 5. Whereas, this distance is lower than the form error for inactive constraint.

### 4.1. Vertex vs. Non-Vertex Solution

A solution $y^{*}$ is said to be vertex if the number of active constraints is greater or equal to n+1 (n is the number of unknowns parameters). If the number of active constraints is strictly less than n+1,$\;y^{*}$ is said to be a non-vertex solution. By definition, a constraint C(y)≥0 is active at $y^{*}$ only if $C\left(y^{*}\right)=0$.

**Figure 6:**
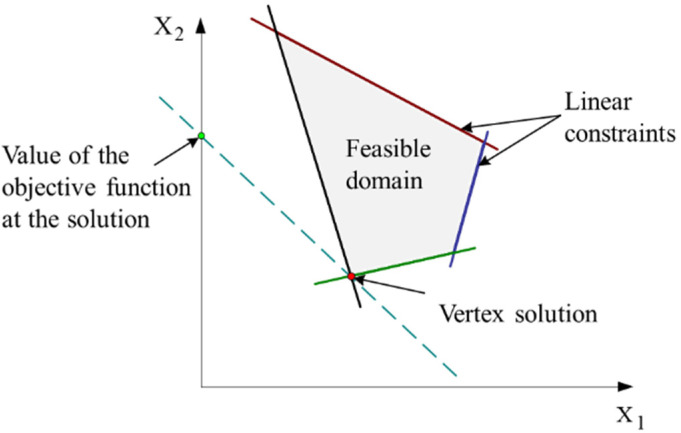
Representation of the vertex solution for the case of a linear programming involving the two variables X_1_ and X_2_.

**Figure 7:**
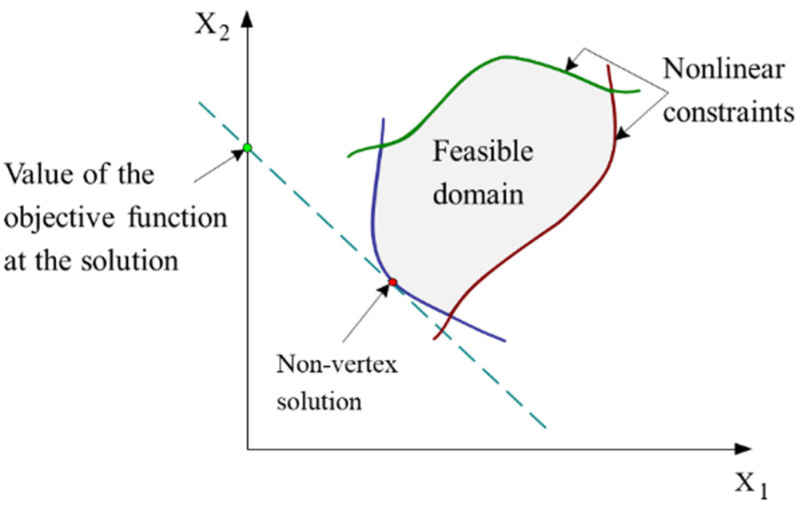
Representation of the non-vertex solution for the case of a nonlinear programming involving the two variables X_1_ and X_2_.

For canonical surfaces, the MZ fitting is almost formulated as a linear programming [26] and the solution could be at some vertex of the feasible domain (Figure 6). For complex surfaces, the solution could be either at some vertex of the feasible domain, or at a face or an edge (non-vertex solution (Figure 7)).

If at $P^{*}$ there can be no binding feasible descent direction (Figure 5), then $P^{*}$ solves the equality constrained sub-problem in (6).

(6) $$\underset{y}{min}\;f(y)\;\mathrm{subject}\;\mathrm{to}\;C_{i}(y)=0,\;\;i\;\epsilon \;I^{*}$$

where $I^{*}$ is the set of active constraint.

Therefore, $P^{*}$ is considered as a local solution of the equality constrained problem (6), in particular when there exist Lagrangian multipliers $\lambda^{*}$ for which ($P^{*},\lambda^{*}$) is a stationary point of the Lagrangian L(x,λ) defined in (7).

(7) $$L\left(x,\lambda \right)=f\left(x\right)-\Sigma_{i\in I^{*}}\lambda_{i}C_{i}\left(x\right)\;\;\;\;\;$$

Considering the first order derivative of Lagrangian L(x,λ), both $\lambda^{*}$ and $P^{*}$ should satisfy the KKT Equation (8).

(8) $$\{\begin{array}{c} \nabla_{x}f\left(P^{*}\right)=\underset{i\in I^{*}}{\sum }\lambda_{i}^{*}\nabla_{x}C_{i}\left(P^{*}\right)\\ C_{i}\left(P^{*}\right)=0,\;\;\;\;i\in I^{*} \end{array}$$

### 4.2. Optimality Conditions

If the number p (belongs to $I^{*}$) of active constraints is less than n (number of unknowns parameters), let Z=z(a) be the n×(n−p) orthogonal complement to $C^{*}$ the matrix of gradients $\nabla_{a}C_{i},\;i\in I^{*}$, so that $Z_{t}C^{*}=0$. Hence, the second order optimality conditions are required.

- The necessary conditions for $P^{*}$ to be a local minimiser are:
  ○  feasibility: $C_{i}\left(p_{i}^{*}\right)\geq 0\;\forall i\in \left\{1,\;\ldots ,N\right\}$
  ○  first order: if $I^{*}$ is the set of active constraints at $p^{*}$, there exist a Lagrangian multiplier $\lambda^{*}$ for which:
    (9) $$\{\begin{array}{c} \nabla_{x}f\left(P^{*}\right)=\underset{i\in I^{*}}{\sum }\lambda_{i}^{*}\nabla_{x}C_{i}\left(P^{*}\right)\\ \lambda_{i}^{*}\geq 0;\;i\in I^{*} \end{array}\;$$
  ○  second order: if p<n, the matrix $Z{\left(P^{*}\right)}^{t}W\left(P^{*},\lambda^{*}\right)Z\left(P^{*}\right)$ is positive semi-definite *(*W is the Hessian matrix of the Lagrangian (7)).

- The sufficient conditions for $P^{*}$ to be a local minimizer under constraint qualification are:
  ○  feasibility: $C_{i}\left(P^{*}\right)\geq 0\;\forall i\in \left\{1,\;\ldots ,N\right\}$
  ○  first order: if $I^{*}$ is the set of active constraints at $P^{*}$, there exist a Lagrange multiplier $\lambda^{*}$ for which:
    (10) $$\{\begin{array}{c} \nabla_{x}f\left(P^{*}\right)=\underset{i\in I^{*}}{\sum }\lambda_{i}^{*}\nabla_{x}C_{i}\left(P^{*}\right)\\ \lambda_{i}^{*}>0;\;i\in I^{*} \end{array}\;$$
  ○  Second order: if p<n, the matrix $Z{\left(P^{*}\right)}^{t}W\left(P^{*},\lambda^{*}\right)Z\left(P^{*}\right)$ is positive definite.

### 4.3. Reference Softgauges Generation for Complex Surfaces: Aspherical Shapes

A method to generate reference softgauges is introduced for the case of complex surfaces when motion parameters are sought. Since the aspherical lenses are rotationally symmetric surfaces, only five motion parameters are unknown$\;x=\left\{T_{X},\;T_{Y},T_{Z},\;\theta_{X},\;\theta_{Y}\right\}$ (translations in X, Y and Z directions as well as rotations around X- and Y-axis), then a non-vertex solution consists of, at most, five contacting points (active constraints). The proposed algorithm includes 7 steps as follows:

Step 1: five points ${\left\{Q_{i}^{*}\right\}}_{i=1,..,5}$ are randomly selected on the nominal aspherical surface (Figure 5). These points represent the orthogonal projections of the contacting points ${\left\{P_{i}^{*}=\left(x_{i}^{*},\;y_{i}^{*},\;z_{i}^{*}\right)\right\}}_{i=1,..,5}$ onto the nominal shape.

Step 2: an index $\alpha_{i}\in \left\{0,1\right\}$ is associated to each point in ${\left\{Q_{i}^{*}\right\}}_{i=1,..,5}$. A point with $\alpha_{i}=1$ represents the orthogonal projection of a contacting point assigned to the lower surface$\;\left({𝒮}^{-}\right)$, i.e., for which $d_{i}\left(Q_{i}^{*},\;P_{i}^{*}\right)=-e$. Those with $\alpha_{i}=0$ are assigned to the upper$\;\left({𝒮}^{+}\right)$ i.e., for which $d_{i}\left(Q_{i}^{*},\;P_{i}^{*}\right)=e$ (e is the desired form error).

Step 3: verify that the gradient vectors of distances with respect to motion parameters $\nabla d_{i}\left(Q_{i}^{*},\;P_{i}^{*}\right)$ are linearly independent. Otherwise, go to step 1. The gradient vectors are calculated using equations given from (11) to (15).

(11) $$\frac{\partial d_{i}}{\partial T_{X}}={\left(-1\right)}^{\alpha_{i}}{\;n}_{i,X}$$

(12) $$\frac{\partial d_{i}}{\partial T_{Y}}={\left(-1\right)}^{\alpha_{i}}{\;n}_{i,Y}$$

(13) $$\frac{\partial d_{i}}{\partial T_{Z}}={\left(-1\right)}^{\alpha_{i}}n_{i,Z}$$

(14) $$\frac{\partial d_{i}}{\partial \theta_{X}}={\left(-1\right)}^{\alpha_{i}}(Q_{i}^{*}\times n_{i}).e_{X}$$

(15) $$\frac{\partial d_{i}}{\partial \theta_{Y}}={\left(-1\right)}^{\alpha_{i}}(Q_{i}^{*}\times n_{i}).e_{Y}$$

where $n_{i}=\left(n_{i,X},\;n_{i,Y},\;n_{i,Z}\right)$ is the normal vector to the nominal shape at the point ${\left\{Q_{i}^{*}\right\}}_{i=1,..,5}$, a×b defines the cross product of the two vectors a and b. $e_{X}$ (resp.$e_{Y}$) is the unit vector with respect to the X-axis (resp. Y-axis).

Step 4: determine Lagrangian multipliers $\lambda^{*}=\left(\lambda_{1}^{*},\ldots ,\lambda_{5}^{*}\right)$ by solving the quadratic programming given in (16).

(16) $$\underset{\lambda }{min}\left|\left|G\lambda -b\right|\right|\;st.\;\lambda \geq 0$$

where G is a 6 × 5 matrix, such that $G=\left(g_{1},\;\ldots ,g_{5}\right)$, $g_{i}^{T}=\left({\left(-1\right)}^{\alpha_{i}}\nabla d_{i}^{T},1\right)$ and $b^{T}=\left(0,0,0,0,0,1\right)$. If ||Gλ−b||>ϵ, where ϵ is a predefined parameter, go to step 1. This step represents the resolution of the equation resulting from KKT conditions of the problem given in (3) cited in [14].

Step 5: determine a nonzero null space vector p such that $G^{T}p=0$.

Step 6: verify that $p_{b}^{T}Hp_{b}>0$ where $p_{b}$ is the vector composed of the first five elements of p and H is the Hessian of the Lagrangian given in (17).

(17) $$H=\overset{5}{\underset{i=1}{\sum }}{\left(-1\right)}^{\alpha_{i}}{\;\lambda }_{i}^{*}\nabla^{2}d_{i}$$

If this condition is not satisfied, then go to step 1. Otherwise, calculate contacting points coordinates by setting $P_{i}^{*}=Q_{i}^{*}+{\left(-1\right)}^{\alpha_{i}}en_{i\;\left(i=6,..,N\right)}$.

Step 7: generate additional random points ${\left\{P_{i}\right\}}_{i=6,..,N}$ such that $P_{i}=Q_{i}+\theta_{i}n_{i}$ with ${\left\{\theta_{i}\right\}}_{i=6,\ldots ,N}$ is a set of randomly selected numbers in the domain [−e, e].

It is worth mentioning that the proposed robust reference softgauges algorithm could work on all complex surfaces, as the only required knowledge is the normal vector with respect to the X-, Y- and *Z*-axis. Furthermore, the developed approach could be applied to continuous nominal shape described by a mathematical model or to discrete high accurate measured dataset.

## 5. Hybrid Trust Region vs. Exponential Penalty Function for Minimax Fitting

### 5.1. Hybrid Trust Region (HTR) Algorithm

The hybrid trust region algorithm consists of performing either the trust region step, line search step or curve search step, according to the specific situation faced at each iteration [8,27,28]. It enables one to avoid having to solve the trust region problem many times. Each iteration relies on obtaining a trust region trial step $d_{k}$ by solving the following quadratic problem (QP) given in (18).

(18) $$\begin{array}{c} min_{\left(d\epsilon {\mathbb{R}}^{n+1}\right)}\frac{1}{2}<d,B_{k}d>+z=M_{k}\left(d,z\right),\;\\ S.t<\nabla f_{i}\left(x_{k},d\right)>-z\leq \phi \left(x_{k}\right)-f_{i}\left(x_{k}\right),\;\;i=1,\ldots ,m\;\;\;\;\;\;\;\;\;\;\;\;\\ d_{k}{}_{\infty }\leq \Delta_{k} \end{array}$$

$B_{k}$ is (n×n) symmetric definite matrix, $\Delta_{k}$ is user-defined coefficient of the domain of the trust region, z is a parameter that depends on the first derivative of φ, meanwhile <.,.> is the dot product. The trust region domain is defined using MZ instead of LS; thus, QP becomes easily solved. The QP in (18) always has a solution, since (0,0) lies inside the feasible domain. This QP could be solved using adapted methods, such as interior point method [25]. If the resulting trust region trial step $d_{k}$ could not be accepted, a corrected step $d_{k}+{\tilde{d}}_{k}$ is determined by solving the QP in (19).

(19) $$\{\begin{array}{c} \min_{\left(\tilde{d}\epsilon {\mathbb{R}}^{n+1}\right)}\frac{1}{2}<d_{k}+\tilde{d},B_{k}\left(d_{k}+\tilde{d}\right)>+\tilde{z}=\tilde{M_{k}}\left(\tilde{d},\tilde{z}\right),\\ S.t<\nabla f_{i}\left(x_{k},\tilde{d}\right)>-\tilde{z}\leq \phi \left(x_{k}+d_{k}\right)-f_{i}\left(x_{k}+d_{k}\right),\;\;i=1,\ldots ,m\\ d_{k}+{\tilde{d}}_{\infty }=\Delta_{k} \end{array}$$

If the corrected $d_{k}+{\tilde{d}}_{k}$ is not accepted in the trust region scheme, then a line search or curve search along $d_{k}$ is performed when $d_{k}$ is a descent direction $r_{k}>0$. If $\left(r_{k}\leq 0\right)$, a step length is sought by performing a curve search that verifies (20).

(20) {ϕ(xk+tkdk+tk2d˜k)≤ϕ(xk)−αtk<dk,Bkdk>α ∈[0,12]

**Figure 8:**
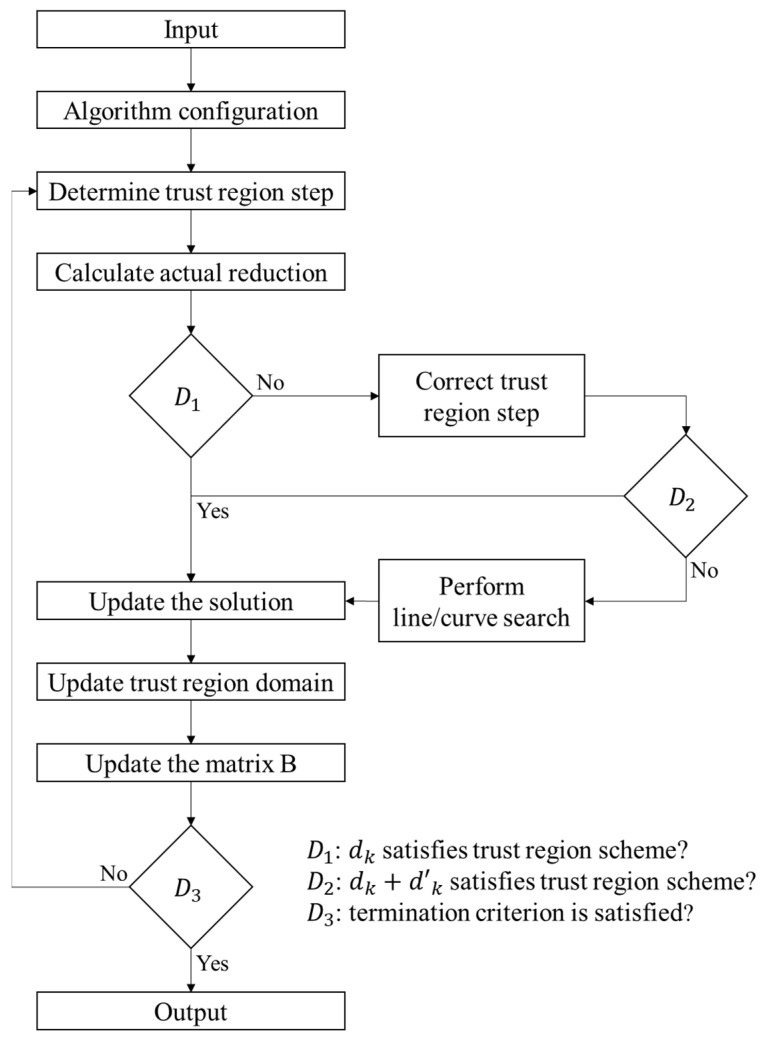
Flowchart of the hybrid trust region (HTR) algorithm.

$d_{k}$ is the solution of (18) and ${\tilde{d}}_{k}$ is the solution of (19). In this case, $\|d_{k}\|\leq \|{\tilde{d}}_{k}\|,\;{\tilde{\;d}}_{k}$ should be taken to be 0. Additional details are discussed in [8,28] and the following flowchart (Figure 8) illustrates how the HTR algorithm performs.

### 5.2. Exponential Penalty Function (EPF) Algorithm

Exponential penalty function is a technique that aims at approximating the non-smooth objective function in the MZ fitting by a parameterized smooth function. The resulting smooth function is optimized using Newton-based methods [23,27,29]. Let (21) be the approximation function, $\left\{d_{i}\left(x\right)\right\}$ are the set of distances between the measured data and the reference surface.

(21) $$\underset{x}{min}\Phi_{MZ}=\underset{1\leq i\leq N}{max}d_{i}\left(x\right)$$

Let $F_{p}$ be the continuously differentiable approximation function and (p>0) the smoothing parameter, as in (22). One can demonstrate the inequality given in (23).

(22) $$F_{p}=\frac{1}{p}Log\left(\overset{N}{\underset{i=1}{\sum }}\exp\left(pd_{i}\left(x\right)\right)\right)$$

(23) $$\forall x\in {\mathbb{R}}^{n},\;\;F\left(x\right)\leq F_{p}\left(x\right)\leq F\left(x\right)+\frac{LogN}{p}$$

When the value of p goes to infinity, $F_{p}$ converges to F. For a given value of the smoothing parameter p, minimisation of $F_{p}$ is carried out through derivative-based methods. Once the optimum of $F_{p}$ is found, p is multiplied by a user-defined coefficient, and a new approximation function is formulated. This process is repeated until the resulting approximation function $F_{p}$ becomes sufficiently close to the original objective function F.

### 5.3. Numerical Validation on Aspherical Shapes and Discussion

**Figure 9:**
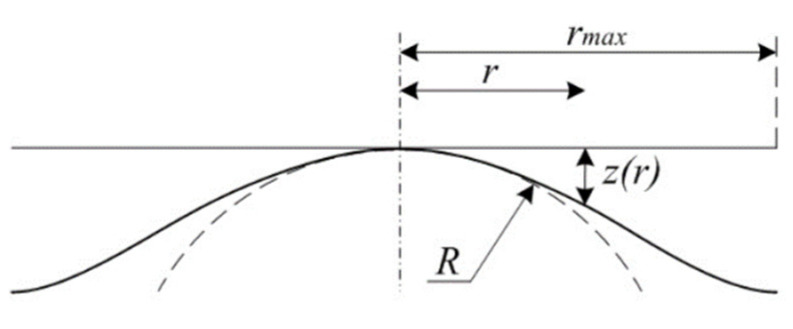
Description of aspherical shapes.

The developed method for reference softgauges generation based on a non-vertex solution is applied for the case of aspherical shapes. An asphere could be defined as a rotationally symmetric surface with a radius of curvature that varies gradually from the centre of the lens (Figure 9). Optical aspherical surfaces are very popular in the domain of optical design regarding their superiority over classical spherical lenses, as well as advancements made in manufacturing techniques [30]. Several formulations could be employed for the description of asphere; however, the most widely used is the one given in ISO 10110-12:2007 [30] called monomial formulation, given in (24). This formulation depends on the sag of the surface parallel to the radial symmetric axis z, radius r, radius of curvature R, conic constant κ and monomial coefficients $a_{2m+4}$.

(24) $$z\left(r\right)=\frac{r^{2}}{R\left(1+\sqrt{1-\left(1+\kappa \right)\frac{r^{2}}{R^{2}}}\right)}+\overset{M}{\underset{m=0}{\sum }}a_{2m+4\;}r^{2m+4}$$

Three configurations are selected, as given in Table 1. For each configuration, a dataset with predefined number of points N={10,404, 50,024, 100,489} and $MZ_{ref}=10^{-4}\;\mathrm{mm}$ are generated to evaluate the performance of the two implemented MZ fitting algorithms (HTR and EPF).

**Figure 10:**
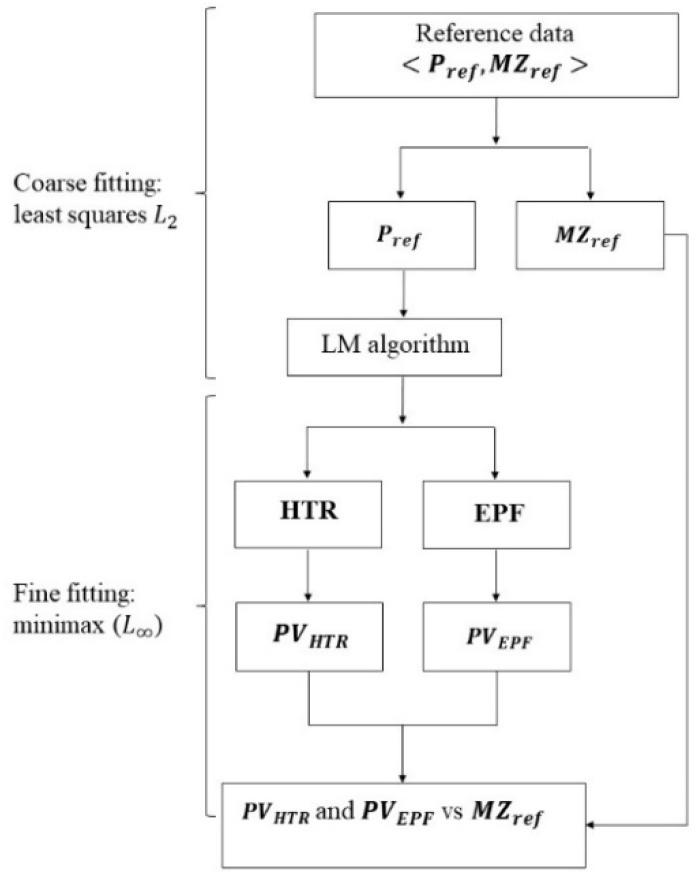
Comparison methodology (LM algorithm: Levenberg–Marquardt algorithm).

The corresponding PV values respectively $PV_{EPF}$ (or *MZ_EPF_*) and $PV_{HTR}$ (or *MZ_HTR_*), as well as execution time, respectively $T_{EPF}$ and $T_{HTR}$, are compared with respect to the methodology presented in Figure 10. Initial data is rotated by angle π/20 around X-axis and π/15 around Y-axis, as well as translated by −1 mm in X-axis, 1 mm in Y-axis and −1 mm in Z-axis. Both HTR and EPF algorithms were implemented on a computer based on Intel Core i7/x64 platform with 16 GB of RAM and a 2.30 GHz processor.

**Figure 11:**
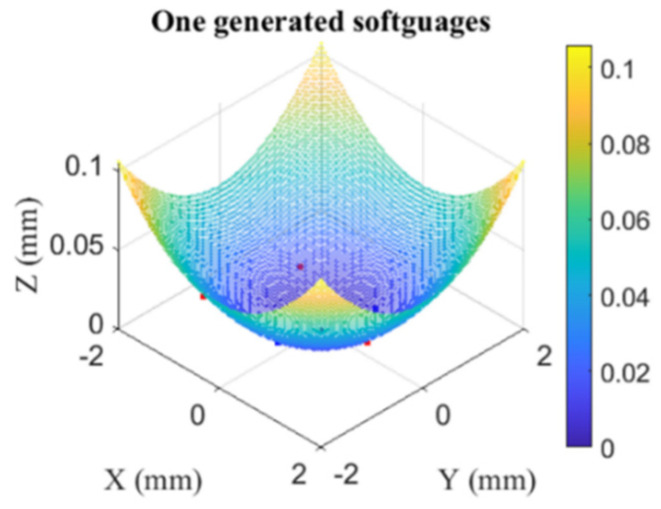
One generated reference softgauges with a non-vertex solution (five contacting points).

**Figure 12:**
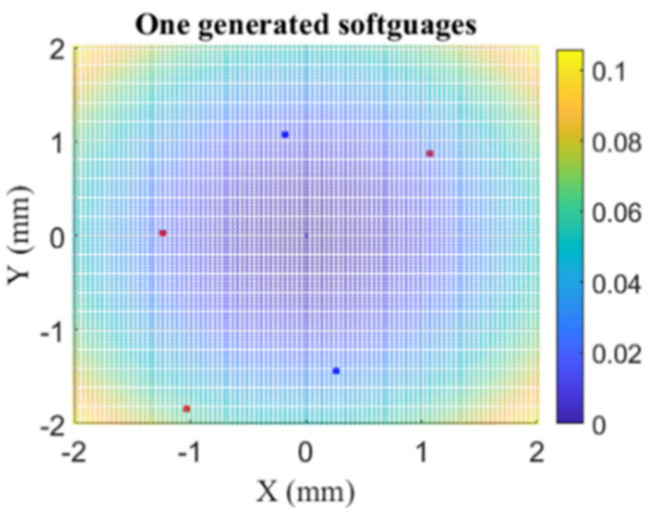
Illustration of the five contacting points (red and blue). Red indicates contacting points for which the form error is equal to *e*. Blue indicates form error equals to −*e*.

Nine reference aspherical softgauges were generated and Figure 11 and Figure 12 illustrate only one generated softgauges with five contacting points. Each generated softgauge was submitted to both HTR and EPF fitting algorithms, and Table 2, Table 3 and Table 4 summarize the returned results of *MZ-MZ_ref_* and the execution time for configurations I, II and III.

According to Table 2, Table 3 and Table 4, it seems that HTR and EPF fitting algorithms return quite similar results of MZ, accurate at the sub-nanometre level, with a noticeable upper hand of HTR when the execution time is regarded. This is due to EPF approximation of the non-smooth objective function in the MZ fitting by one parametrized smooth function. Then, the applied Newton method requires the computation of the Hessian matrix, which is proportional to the number of points in the softgauges. Moreover, the accuracy of the obtained descent direction is not always guaranteed. Hence, corrections must be brought to the Hessian matrix whenever needed.

Regarding the HTR algorithm, the matrix $B_{k}$ is chosen to be symmetric positive definite while setting up the QP, through Powell modification BFGS (Broyden–Fletcher–Goldfarb–Shanno) formula. Therefore, there is no need to compute the second order derivation terms, which considerably reduces the execution time.

## 6. Difficulty Degree and Performance Measure

Measuring an algorithm quality could be established by setting a framework, including: (1) the preconditions for quality measurement, (2) the analysis strategy of the meaningfulness of quality measures, as well as (3) the interpretation and use of the measured values [31].

The degree of difficulty was discussed by Cox and Harris in [32]. It aims at defining a quantity associated to each dataset that indicates at which level the generated reference softgauges challenge the algorithm under test. Thus, the methodology of testing metrology MZ fitting algorithms consists of generating datasets with increasing difficulty number. The output of the algorithm under test is assessed using a performance measure on each set. Therefore, the degree of difficulty could be sought as the difficulty to converge to a global optimum. Usually, the selected feature could help in predicting the difficulty of the MZ fitting problem. It regroups: (1) the nature of the solution, (2) the number of points affecting tremendously both execution time as well as the accuracy, (3) the initial position of the measured data compared to the final position.

The suggested degree of difficulty denoted by λ is given in (25).

(25) $$\lambda =\beta_{1}V+\beta_{2}\frac{N}{N_{0}}+\beta_{3}\frac{\Theta }{\Theta_{0}}$$

where $\beta_{1}$, $\beta_{2}$ and $\beta_{3}$ are user-defined parameters, such that: $\overset{3}{\underset{i=1}{\sum }}\beta_{i}=1$, V is a binary variable that indicates whether the set of data is vertex or non-vertex, N is the number of the data points in the set and $N_{0}$ is the maximum number of points that could be handled by the algorithm under test. Θ is the measure of the initial position. $\Theta_{0}$ is the estimation of the maximum initial alignment, calculated by taking the norm of the vector of maximum permissible translation and rotation.

The performance measure For MZ fitting could be considered as the difference between the minimum zone value returned by the algorithm under test and the reference value. When execution time is involved, the performance measure could combine both result accuracy and execution time. In addition, the heuristic/deterministic aspect of the algorithm under test should be considered, because deterministic algorithms are more appreciated for metrology software. The latter requirement could be assembled in a sample of performance measure denoted η given in (26).

(26) $$\eta =\alpha_{1}f_{1}\left(E,\lambda \right)+\alpha_{2}f_{2}\left(T,\lambda \right)+\alpha_{3}f_{3}\left(\Delta ,\lambda \right)$$

where$\;\alpha_{1}$, $\alpha_{2}$ and $\alpha_{3}$ are user-defined parameters, such that: $\overset{3}{\underset{i=1}{\sum }}\alpha_{i}=1$. The expression of the functions $f_{1}$, $f_{2}\;$and $f_{3}$ are given in (27)–(29), respectively.

(27) $$f_{1}\left(E,\lambda \right)=\{\begin{array}{c} \frac{E_{0}\left(\lambda \right)}{E},\;if\;E>E_{0}\left(\lambda \right)\\ 1\;\;\;,\;\mathrm{otherwise} \end{array}$$

(28) $$f_{2}\left(T,\lambda \right)=\{\begin{array}{c} \frac{T_{0}\left(\lambda \right)}{T},\;if\;T>T_{0}\left(\lambda \right)\\ 1\;\;\;,\;\mathrm{otherwise} \end{array}$$

(29) $$f_{3}\left(\Delta ,\lambda \right)=\{\begin{array}{l} 0\;\;\;,\;\mathrm{if}\;\mathrm{heuristic}\\ 1\;\;\;,\;\mathrm{if}\;\mathrm{deterministic} \end{array}$$

$E_{0}$ and $T_{0}$ are the recommended error and execution time of each value of the number of difficulty, while *T* and *E* are the actual execution time and the *MZ* value.

**Figure 13:**
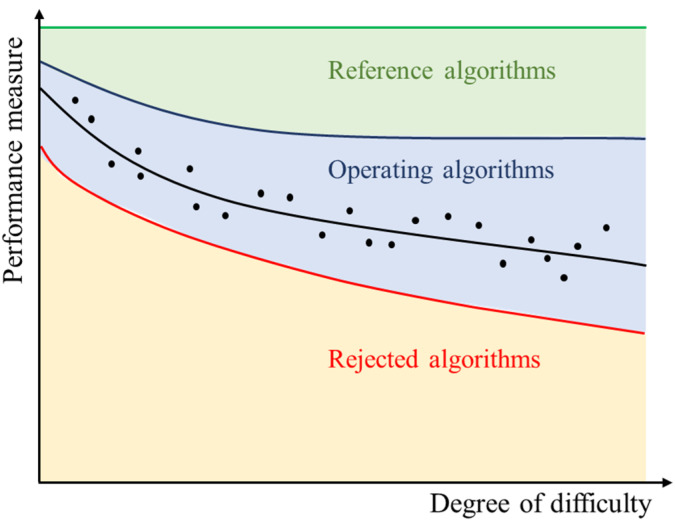
Performance measure vs. of degree of difficulty for a given algorithm under test.

Figure 13 illustrates the performance of a given *MZ* fitting algorithm under test, as well as the acceptance domain for reference algorithms and operating ones. Since an algorithm of reference and an operating one do not have the same operational requirements, these two types of algorithms present two different characteristics in the degree of difficulty and performance measure domain.

## 7. Analysis of the Results and Discussion

With the aim of defining the weights associated to the samples of performance measure and the degree of difficulty, a survey was conducted involving 45 engineers and researchers from industrial and academic fields, NMIs (National Metrology Institutes) and others.

The survey is based on questions regarding how important it is that an algorithm gives accurate results, runs in short time and returns deterministic or heuristic results. Then, the weights $\alpha_{1}$, $\alpha_{2}$ and $\alpha_{3}$ were estimated based on the survey’s answers (Table 5).

EPF is considered as an operating algorithm and the determination of $\beta_{1},\;\beta_{2}$ and $\beta_{3}$ is given as follow: $\beta_{1}=0.5$, $\beta_{2}=0.5$ and $\beta_{3}=0\;$(since the coarse fitting has taken place before proceeding to fine fitting). The recommended execution time and the form error were determined through $L_{2}$- fitting (LS).

The function that determines the accepted limits could be formulated as a convex combination of the form tb+(1−t)a, where a is the performance measure of the algorithm under test for the first set, b=a−ϵ, ϵ is the user-defined coefficient and t∈[0,1].

**Figure 14:**
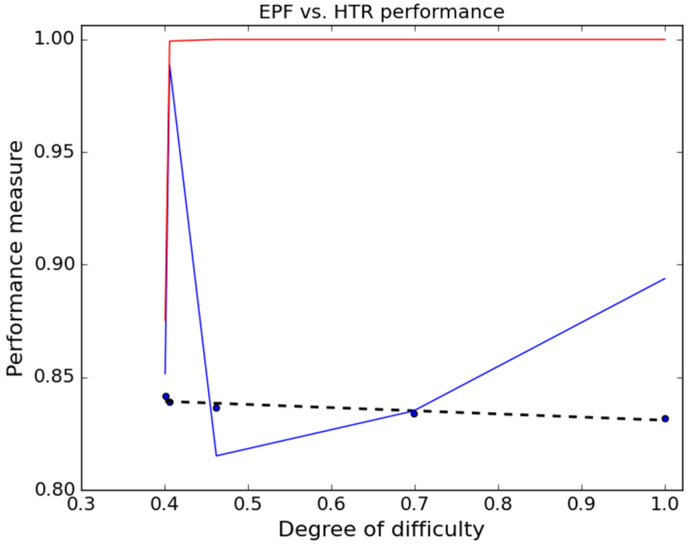
EPF vs. HTR performance in blue and red (respectively). In black: the fitted line defines the accepted limits of the performance measure.

The obtained performance measure results are presented in Figure 14. Therefore, HTR is as stable as an algorithm of reference could be. This is due to its accurate $MZ_{HTR}$ values and the execution time that was not affected by the number of points in the dataset. EPF is unstable with an increased degree of difficulty, as it is sensitive to the number of points in the dataset.

Nevertheless, vertex or non-vertex solution cannot be the only concern when constructing reference softgauges for MZ fitting. There are still some requirements needed before proceeding to softgauges generation; in particular, the scope and characteristics of the algorithm under test must be clearly identified. Then the abilities claimed by the algorithm should be determined so that task-specific data points are generated and the algorithm to test is not “disfavoured”.

These characteristics may include:

- **Uniqueness of the solution:** generated reference softgauges must have a unique associated reference solution [33]. For fitting problems, reference softgauges with two different “substitute geometry” will be problematic, especially if these geometries will be taken as datum. These situations must be avoided when generating reference softgauges.
- **Number of points:** the number of points contained in the reference softgauges must not exceed the maximum number of points that could be handled by the algorithm.
- **Initial alignment:** initial position of measured data highly affects the performance of MZ fitting algorithms. Some algorithms could only perform if the input data are close to the solution position.
- **Error-free:** validation of metrology MZ fitting algorithms must be performed in the perfect operator approximation. This means that measuring errors (or errors from any origin) must not be embedded in constructed data. Adding measuring errors to the reference softgauges might induce some difficulties, since we cannot tell whether inaccuracy results from measurement or processing.
- **Stability:** a small perturbation in the designed data must not affect the reference value of the measurand. The analysis of an MZ fitting algorithm stability was related to the values of Lagrangian multipliers [11,34].

- **Uncertainty:** it should be associated to reference softgauges. Considering the data coordinates $\left(x_{i}^{*},\;y_{i}^{*},z_{i}^{*}\right)$ and targeted form error $e_{t}$ expressed in double precision as well as the LICQ and dual feasibility conditions satisfied, the actual value of form error e is given in (30).

(30) $$e=\sqrt{{\left(x_{i}-x_{i}^{*}\right)}^{2}+{\left(y_{i}-y_{i}^{*}\right)}^{2}+{\left(z_{i}-z_{i}^{*}\right)}^{2}}$$

with $x_{i}=x_{i}^{*}+e_{t}n_{i,X}$, $y_{i}=y_{i}^{*}+e_{t}n_{i,Y}$ and $z_{i}=z_{i}^{*}+e_{t}n_{i,Z}$

Uncertainties of $x_{i}^{*},\;y_{i}^{*}\;\mathrm{and}\;z_{i}^{*}$ and $e_{t}$ depend on the accuracy of the machine architecture, while uncertainties of $x_{i},\;y_{i},\;z_{i}$, $n_{i,X}$, $n_{i,Y}$ and $n_{i,z}$ could also be calculated analytically using propagation rules. Thus, the uncertainty of the actual form error e denoted by u(e) could be estimated.

## 8. Conclusions

In this paper, the generation of reference softgauges dedicated to the assessment of MZ fitting algorithms of complex shapes is provided. The developed robust approach is based on satisfying KKT first and second order optimality conditions before inferring reference softgauges with non-vertex solutions. It could be applied to continuous nominal shape described by a mathematical model or to discrete high accurate measured dataset.

The implemented approach based on a non-vertex solution was adopted and investigated for the case of aspherical shapes. Nine reference softgauges were generated with predefined number of points and $MZ_{ref}=10^{-4\;}\mathrm{mm}$. The reference softgauges were submitted to two implemented MZ fitting algorithms (HTR and EPF). Results show the conformance of the returned values of $MZ_{HTR}$ and $MZ_{EPF}$ with the reference measurand ones $MZ_{ref}$. However, the performance of the HTR fitting algorithm in terms of execution time is noticeable in comparison to the EPF one.

Two metrics were introduced to define the performance measure in the function of the degree of difficulty to measure the performance of an MZ fitting algorithm. As these two metrics contains some arbitrary variables, a survey based on a number of industrial and academic professionals has allowed us to determine these weights. An application on HTR and EPF fitting algorithms was carried out such as to measure their performances. The given results reveal that EPF performs poorly when the number of points is higher, especially when the execution time is taken into consideration.

Requirements of the generated reference softgauges were also discussed in this article. In fact, generated softgauges must be aligned with characteristics of the MZ fitting algorithm under test. Data points’ extraction, number of points, stability of the solution, etc. must be taken into account when generating softgauges. Most of the considered requirements need further research. Therefore, future work will mainly cover original methods for assessing the stability of generated softgauges, in particular for the case of complex shapes.

## Figures and Tables

**Table 1:** Nominal values of the aspheric shapes parameters.

Configuration	R (mm)	*k*	*a*_4_ (mm^−3^)	$a_{6}\;\left(mm^{-5}\right)$	$a_{8}\;\left(mm^{-7}\right)$	$a_{10}\;\left(mm^{-9}\right)$
I	19.79	−0.9	$-1.5\times 10^{-17}$	$-7.55\times 10^{-18}$	$-3.77\times 10^{-18}$	$-1.88\times 10^{-19}$
II	8.88	−0.8	$-1.9\times 10^{-12}$	$-9.72\times 10^{-13}$	$-4.86\times 10^{-13}$	$-2.43\times 10^{-13}$
III	4.14	−0.9	$-4.1\times 10^{-12}$	$-2.08\times 10^{-12}$	$-1.04\times 10^{-12}$	$-5.21\times 10^{-13}$

**Table 2:** Values of MZ-MZ_ref_ and execution time (s) for HTR and EPF (configuration I).

*N*	$MZ_{HTR}-MZ_{Ref}\;\left(mm\right)$	$MZ_{EPF}-MZ_{Ref}\;\left(mm\right)$	$T_{HTR\;}\left(s\right)$	$T_{EPF}\;(s)$
10,404	1.78 × 10^−16^	4.51 × 10^−16^	12.1	61.91
50,024	1.71 × 10^−16^	4.78 × 10^−16^	24.5	135.71
100,489	1.64 × 10^−16^	4.92 × 10^−16^	41.51	226.96

**Table 3:** Values of MZ-MZ_ref_ and execution time (s) for HTR and EPF (configuration II).

*N*	$MZ_{HTR}-MZ_{Ref}\left(mm\right)$	$MZ_{EPF}-MZ_{Ref\;}\left(mm\right)$	$T_{HTR}\;\left(s\right)$	$T_{EPF\;}(s)$
10,404	1.41 × 10^−15^	3.08 × 10^−15^	4.27	36.50
50,024	3.78 × 10^−16^	4.78 × 10^−16^	10.78	157.71
100,489	6.73 ×1 0^−15^	6.78 × 10^−15^	20.52	255.06

**Table 4:** Values of MZ-MZ_ref_ and execution time (s) for HTR and EPF (configuration III).

*N*	$MZ_{HTR}-MZ_{Ref\;}\left(mm\right)$	$MZ_{EPF}-MZ_{Ref\;}\left(mm\right)$	$T_{HTR}\;\left(s\right)$	$T_{EPF\;}(s)$
10,404	1.15 × 10^−16^	2.14 × 10^−15^	35.88	200.16
50,024	4.78 × 10^−16^	1.78 × 10^−16^	42.35	240.45
100,489	9.86 × 10^−15^	1.05 × 10^−14^	50.03	274.75

**Table 5:** Estimation of the weights based on the survey answers.

	Associated Weights	
	Reference algorithm	Operating algorithm
$\alpha_{1}$	0.41	0.4
$\alpha_{2}$	0.24	0.28
$\alpha_{3}$	0.35	0.32

## Data Availability

The data presented in this study are available on request from the corresponding author.
